# The role of perceptual and action effector strength of graphs and bases of mathematical metaphors in the metaphorical processing of mathematical concepts

**DOI:** 10.3389/fpsyg.2023.1178095

**Published:** 2023-08-09

**Authors:** Omid Khatin-Zadeh, Danyal Farsani, Jiehui Hu, Fernando Marmolejo-Ramos

**Affiliations:** ^1^School of Foreign Languages, University of Electronic Science and Technology of China, Chengdu, China; ^2^Norwegian University of Science and Technology, Trondheim, Norway; ^3^University of South Australia Online, Adelaide, SA, Australia

**Keywords:** action effector strength, mathematical concepts, mathematical metaphors, perceptual strength, embodiment

## Abstract

Metaphors that describe an abstract concept in terms of a motion concept are widely used to enhance our understanding of abstract concepts. These metaphors are used not only in our daily language but also in learning mathematics. As an example, in the process of understanding the abstract representation of a mathematical concept, a graphical representation may play the role of a mediatory domain. This graphical representation could have a high degree of perceptual and action effector strength. This is particularly the case when a gestures (as a motion) is used to depict the graphical representation. After looking at this example, we discuss perceptual and action effector strength of the base domains of several mathematical metaphors that describe mathematical concepts in terms of spatial and motion concepts. Then, based on the data in the Lancaster Sensorimotor Norms, it is suggested that high degrees of perceptual and action effector strength of the base domains of these metaphors play an important role in the grounding of abstract mathematical concepts in the physical environment.

## 1. Introduction

Metaphors, the ubiquitous feature of language, are tools for better expressing our ideas and communicating more efficiently. When it is difficult for us to literally talk about our ideas, we use metaphors to offer a clearer picture of what we are talking about. Metaphors are also a way for acquiring a better understanding of others’ ideas. Lakoff and Johnson (2003) define metaphor as a tool for understanding one concept (usually an unfamiliar abstract one) in terms of another concept (usually a familiar concrete one). The familiar concrete concept is the base and the abstract one is the target of the metaphor. Metaphors are used not only in daily language to talk and communicate about ordinary subjects, but also in scientific discussions to talk about highly technical subjects, such as mathematical ideas (e.g., Núñez and Lakoff, 1998; Lakoff and Núñez, 2000; Marghetis and Núñez, 2013).

An important question about the role of metaphors in enhancing mathematical thought is how metaphors help us to acquire a deeper understanding of mathematical concepts. It has been suggested that using metaphors allows us to employ a wider range of cognitive resources in the process of understanding and learning (Khatin-Zadeh et al., 2021b; Khatin-Zadeh, 2022; Khatin-Zadeh et al., 2023b). This argument is based on the strong version of embodied cognition, according to which the same neural networks and cognitive resources that are involved in the processing of the base of a metaphor are employed to process the target of the metaphor. The strong version of embodiment holds that conceptual representations are primarily formed through sensorimotor systems and processes (e.g., Glenberg et al., 2008; Lakoff, 2008; Connell and Lynott, 2016), while the weaker versions assume a partial role for sensorimotor, emotional, and modality-independent systems (e.g., Binder and Desai, 2011; Hauk and Tschentscher, 2013; Lambon-Ralph, 2013; Zwaan, 2014; for a review, see Khatin-Zadeh et al., 2021a). For example, when abstract numbers are metaphorically described in terms of spatial concepts, the cognitive resources that are involved in the processing of spatial concepts are employed to process abstract numbers. In this metaphorical description, abstract numbers are understood in terms of highly concrete spatial concepts. In this paper, we intend to provide a more comprehensive answer for the question of how metaphors enhance our understanding of mathematical concepts. To answer this question, we first discuss metaphorical description of mathematical functions in terms of graphs in the Cartesian coordinate system as one category of mathematical metaphors.

## 2. Metaphorical description of mathematical functions in terms of graphs

The description of mathematical concepts such as a function through graphs in a Cartesian plane or in a three-dimensional Cartesian coordinate system is one category of mathematical metaphors that a highly abstract mathematical concept can be grounded in concrete concepts through the sensory-motor system. A recent work has suggested that not only mathematical concepts but also ordinary concepts can be processed in terms of embodied graphs (Woodin et al., 2022). Transforming abstract mathematical concepts and problems into visual representations is a common strategy in mathematics. When a mathematical concept is represented by a graph in a Cartesian plane, its behavior can clearly be examined. Here, a mathematical concept can be grounded though visual system as graphs are highly imageable.

It has been proposed that the process of understanding a mathematical function through a curve, which is the graphical representation of that function on the plane of a Cartesian coordinate system, involves the activation of the motor system, as a curve can be conceived as the path of a moving object (fictive motion of an object) on a plane (Khatin-Zadeh et al., 2022a). This proposal is supported by findings of studies suggesting that processing a fictive motion involves mental simulation of an action and activation of the motor system (e.g., Matlock, 2004, 2006; Núñez et al., 2006; Matlock et al., 2011). Based on such findings, Matlock (2010) argues that processing a fictive motion is accompanied by a fleeting sense of motion (see also, Blomberg and Zlatev, 2014). As mentioned, the strong version of embodiment (Gallese and Lakoff, 2005) argues that even the experience of looking at a moving object may involve the activation of the motor system. Therefore, it can be said that perceiving and processing the curve of a function could involve simulating the experience of scanning the path of a moving object on that curve. Even the observer could simulate the experience of her/his movement on the curve. According to Barsalou (2008, 2009), simulating an experience is a process through which perceptual, motoric, and introspective states involved in that experience are re-enacted. Simulating the process of scanning and also simulating the process of movement may involve the engagement of the motor system. In both cases, the motor system may be actively employed to process the function and the curve, which is the visual representation of that function.

The role of the visual and motor systems in the understanding of mathematical concepts that are described in terms of visual representations is supported by some neuroimaging evidence suggesting that even the experience of looking at graphical representations could activate the motor system (e.g., James and Gauthier, 2006; Gallese and Sinigaglia, 2011; Longcamp et al., 2011). In one of these studies, Umilta' et al. (2012) found that the cortical motor system is activated during the processing of static abstract works of art. Another EEG study (Sbriscia-Fioretti et al., 2013) examined the activation of sensorimotor cortical circuits when participants were looking at paintings with marked traces of brushstrokes. Results of this study showed that premotor and motor cortical areas were activated when participants were observing the paintings. Therefore, it can be suggested that the activation of the motor system could take place when an abstract mathematical concept is transformed into or is represented in terms of a graphical representation.

Many abstract continuous mathematical concepts can be represented by continuous graphical representations in a Cartesian plane. Here, the abstract representation and the graphical representation, which is highly concrete, are isomorphic with each other. That is why one representation can be understood in terms of another representation. As mentioned, processing the graphical representation of a mathematical concept may involve the activation of the motor system, as we process the graphical representation through simulating the experience of looking at a moving object on that graph. For example, the curve of a function in a Cartesian plane, which is the graphical representation of that function, can be processed through simulating the experience of looking at a moving object on that curve. Even the observer may simulate the experience of scanning the curve from a starting point to an ending point. In the process of transforming the algebraic representation of a function into a curve in the Cartesian plane, gestures can play a supporting role in showing the movement of an object on that curve. In this way, the sensory-motor system is employed to ground the algebraic function through sensory-motor system. The curve functions as a highly perceivable mediatory domain through which the less perceivable algebraic representation of the function is understood. The gestures that are used to describe the movement of an object on the curve play the role of a mediatory channel.

Representing an abstract mathematical concept in terms of a graphical representation is an example of mathematical metaphors in which a mathematical concept is transformed into a representation with a high degree of perceptual strength. Giving perceptual strength to abstract mathematical concepts may take place through other types of mathematical metaphors. In the following sections, we discuss several of such metaphors.

## 3. Perceptual and action effector strength of the base of a metaphor

As mentioned, Lakoff and Johnson (2003) argue that metaphors are used to describe a less familiar abstract domain in terms of a more familiar concrete domain. An intriguing question is how our conceptual system divides concepts into some that are more concrete and some that are less concrete: how do we come up with the ability to realize degrees of concreteness/abstractness of concepts? Crutch and Jackson (2011) argue that abstractness/concreteness is not a binary but a graded relationship. Therefore, the degree of abstractness/concreteness may range from the extreme point of absolute abstractness to the extreme point of absolute concreteness. In other words, there is not a clear dichotomy between abstract and concrete concepts (Guan et al., 2013; Borghi et al., 2017). Degrees of perceptual and action effector strength are closely related to degree of concreteness. Several studies have been conducted to examine degrees of perceptual strength of concepts (e.g., Filipović Đurđević et al., 2016; Speed and Majid, 2017; Miklashevsky, 2018; Chedid et al., 2019; Chen et al., 2019; Miceli et al., 2021). These studies tried to measure the degrees of perceptual and action effector strength of concepts across sensorial domains (touch, hearing, smell, taste, and vision). Findings from these studies have indicated that a given concept may have higher perceptual ratings in a certain sensory modality.

A comprehensive study (Lynott et al., 2019) examined the perceptual strength of 40,000 English concepts in six modalities (touch, hearing, smell, taste, vision, and interoception) and action strength in five action effectors (mouth/throat, hand/arm, foot/leg, head, and torso). Based on the results of this study, a concept may have a strong sensorial dimension in one specific modality or a strong action effector dimension in one part of the body. For example, the concepts of ‘yellow’ and ‘star’ have strong visual dimensions, while they have weak haptic dimension as they cannot be touched. To take another example, the concept of ‘rhythm’ is strong in auditory dimension, while it is weak in haptic, gustatory, olfactory, and visual dimensions. Lynott et al. (2019) found that some concepts have a strong action effector dimension. For example, the concepts of ‘bagel’ and ‘chew’ have strong mouth action dimension. The concepts of ‘running’, ‘climbing’, and even ‘bath’ have a strong foot action dimension. Several methods have been suggested for obtaining a single value that shows the degree of perceptual strength of concepts on the basis of perceptual strength values in various dimensions (e.g., Connell and Lynott, 2016; Filipović Đurđević et al., 2016; Lynott et al., 2019). Although a distinction has been made between degree of concreteness and degree of perceptual and action strength, these measures are closely correlated with one another. It can be suggested that perceptual strength and action strength are key features of the base domain of a metaphor. If the base domain of a metaphor has a strong perceptual and action strength, the process of grounding the target domain will be facilitated. In the following two sections, we try to answer the main question of the paper on the basis of the role of perceptual and action effector strength of the base of a metaphor in the process of employing a wider range of sensorimotor resources. We do this by discussing perceptual and action effector strengths of concepts that are used to metaphorically describe the mathematical concepts of number, addition, subtraction, and limit of function.

## 4. Perceptual and action effector strength in metaphorical description of numbers, addition, and subtraction

The perceptual and action strength of the base domain could be particularly important in the metaphorical description of mathematical concepts, which is a special type of transforming one representation of a concept into another representation. Many fundamental mathematical concepts are metaphorically understood in terms of spatial and motion concepts (Farsani et al., 2022). Numbers are understood as points on a horizontal line in the three-dimensional space. Large and positive numbers are metaphorically associated with right space or rightward movements, while small and negative numbers are associated with left space and leftward movements (e.g., Dehaene et al., 1990, 1993; Fischer et al., 2003; Daar and Pratt, 2008; Wood et al., 2008). Winter et al. (2013) found that people tend to gesture rightward when talking about large numbers and leftward when talking about small numbers. Numbers are also metaphorically understood as points on a vertical line in the space. Large and positive numbers are metaphorically understood in terms of upper space or upward movement, while small and negative values are understood in terms of lower space and downward movement (Winter et al., 2015; Sixtus et al., 2019). Some studies conducted on vertical, diagonal, and radial associations of numbers have found that small numbers are metaphorically associated with lower, lower left, and near space, while larger numbers are associated with upper, upper right, and far space, respectively (e.g., Grade et al., 2013; Göbel, 2015; Winter et al., 2015; Hesse and Bremmer, 2017). Results obtained by Winter et al. (2013) demonstrated that when people use vertical dimension to talk about numbers, they tend to gesture upward when talking about large numbers and downward when talking about small numbers. Furthermore, it has been found that people tend to point to right space after solving an addition problem and to left space after solving subtraction problems (Pinhas and Fischer, 2008; Pinhas et al., 2014). A study conducted by Masson and Pesenti (2014) suggested that solving addition problems may induce an attentional shift to the right and solving subtraction problems may induce an attentional shift to the left. These suggest that the arithmetic operations of addition and subtraction are metaphorically understood in terms of rightward and leftward movements, respectively. In these metaphorical descriptions, numbers and arithmetic operations among numbers are understood in terms of spatial and motion concepts.

According to the data that have been provided in the Lancaster Sensorimotor Norms (Lynott et al., 2019), spatial and motion concepts have high degrees of perceptual and action effector strength. In the metaphorical description of numbers, addition, and subtraction, some spatial and motion concepts such as MOVEMENT, UP, UPWARDS, DOWN, RIGHTWARDS, RIGHT, LEFT, and DIRECTION are involved. Even in elementary mathematics textbooks, these basic concepts are widely used to describe some fundamental mathematical concepts. The use of these concepts in metaphorical description of mathematical concepts have been discussed in many past works (Fischer et al., 2003; Pinhas and Fischer, 2008; Alibali and Nathan, 2012; Winter et al., 2013, 2015; Masson and Pesenti, 2014; Pinhas et al., 2014; Göbel, 2015; Sixtus et al., 2019; Khatin-Zadeh and Yazdani-Fazlabadi, 2023; Khatin-Zadeh et al., 2023a). In fact, such metaphorical descriptions of fundamental mathematical concepts help students acquire a tangible and grounded understanding of mathematical concepts. These concepts are the constituents of the domains in which the abstract concepts of number, addition, and subtraction are metaphorically understood. We revisited Lynott et al.’s (2019) database and extracted perceptual and action effector strength ratings of some spatial and motion concepts that are used to metaphorically describe the concepts of NUMBER, ADDITION, AND SUBTRACTION. This database is available at https://embodiedcognitionlab.shinyapps.io/perceptual_norms/. The concepts of interest, here in uppercase, were typed in the ‘text input’ field (thus the option ‘text input’). The field ‘data choice’ shows the property of interest, and the field ‘statistics’ enables to output the mean ratings (on a scale from 0 [not experienced at all with that sense/action] to 5 [experienced greatly with that sense/action]) of each concept’s sensorimotor, perceptual, and action properties. Tables 1, 2 show degrees of perceptual and action effector strength of these concepts. Nonparametric multiple contrast tests for repeated measures (given that the same participants rated items in each category) were performed to evaluate potential pairwise significant differences (Noguchi et al., 2020). The adjusted *p*-values of differences of interest are reported along with estimated relative effects (*RE*) based on global rankings of the variables compared (*RE*s range from 0 [no effect] to 1 [large effect]). All other possible pairwise comparisons can be found in the Supplementary files at https://figshare.com/account/items/22794788/edit.

**Table 1:** Norms of perceptual strength of some spatial and motion concepts used to metaphorically describe numbers, addition, and subtraction (Lynott et al., 2019).

Modality	Auditory	Gustatory	Haptic	Interoceptive	Olfactory	Visual	*M* (SD)
Concept							
Movement	1.71	0.00	1.71	2.41	0.00	3.88	1.62 (1.48)
Rightwards	0.93	0.21	0.50	0.57	0.21	2.93	0.89 (1.03)
Upwards	0.65	0.24	0.47	1.18	0.12	3.71	1.06 (1.35)
Direction	1.53	0.21	0.47	1.05	0.16	3.63	1.18 (1.31)
Right	1.54	0.13	0.71	2.04	0.25	2.83	1.25 (1.07)
Left	0.82	0.06	1.71	1.12	0.06	2.82	1.10 (1.06)
Up	0.60	0.00	1.33	2.07	0.00	4.13	1.36 (1.58)
Down	1.22	0.28	0.61	2.61	0.28	3.28	1.38 (1.28)
M (SD)	1.07 (0.44)	0.12 (0.11)	0.86 (0.56)	1.54 (0.75)	0.13 (0.10)	3.47 (0.52)	

The last column and row report the means (M) of the means and their standard deviations (SD; in brackets).

**Table 2:** Norms of action effector strength of some spatial and motion concepts used to metaphorically describe numbers, addition, and subtraction (Lynott et al., 2019).

Action effector	Foot	Hand	Head	Mouth	Torso	*M* (SD)
Concept						
Movement	4.53	4.42	3.32	2.21	3.74	3.64 (0.94)
Rightwards	1.30	1.90	1.85	1.25	1.20	1.50 (0.34)
Upwards	1.20	1.55	2.05	0.45	0.40	1.13 (0.71)
Direction	1.76	2.38	3.10	1.57	0.91	1.94 (0.83)
Right	1.20	2.50	2.35	1.30	0.70	1.61 (0.78)
Left	1.90	2.32	2.47	0.26	0.58	1.51 (1.02)
Up	2.55	3.05	2.70	1.65	2.15	2.42 (0.54)
Down	0.75	0.80	1.75	0.65	0.55	0.90 (0.48)
M (SD)	1.76 (1.19)	2.24 (1.07)	2.42 (0.54)	1.12 (0.64)	1.23 (1.08)	

The last column and row report the means (M) of the means and their standard deviations (SD; in brackets).

In most cases, these concepts have high degrees of perceptual and action effector strength. Among norms of sensorimotor strength, norms of visual modality showed larger effects than others (*RE_visual_* = 0.91, *RE_interoceptive_* = 0.65, *RE_auditory_* = 0.58, *RE_haptic_* = 0.50, *RE_olfactory_* = 0.17, and *RE_gustatory_* = 0.16. All five pairwise comparisons with this modality were *p* < 0.001). Among action effector strength, norms of hand and head showed larger effects than others (*RE_head_* = 0.73, *RE_hand_* = 0.64, *RE_foot_* = 0.48, *RE_mouth_* = 0.31, and *RE_torso_* = 0.31. Out of the four pairwise differences including head, that between head-mouth was significant, *p* < 0.007 and out of the four pairwise differences including hand, those between hand-torso, and hand-mouth were significant, *p* < 0.05). Therefore, these elements seem to play a more critical role in grounding the abstract mathematical concepts of number, addition, and subtraction in the physical environment through metaphorical descriptions.

Tables 3, 4 show norms of perceptual and action effector strength of the concepts of number, addition, and subtraction. Interestingly, among norms of perceptual strength, norms of visual modality showed larger effects than others (*RE _visual_* = 0.91, *RE_auditory_* = 0.75, *RE_haptic_* = 0.56, *RE_interoceptive_* = 0.35, *RE_gustatory_* = 0.27, and *RE_olfactory_* = 0.13. All five pairwise comparisons with this modality were *p* < 0.007). Among action effector strength, norms of hand and head showed larger effects than others (*RE_head_* = 0.90, *RE_hand_* = 0.61, *RE_mouth_* = 0.56, *RE_torso_* = 0.26, and *RE_foot_* = 0.15. All pairwise comparisons including head were significant, *p* < 0.05. In the case of hand, only the pairwise comparison hand-mouth was not significant, *p* = 0.92). Therefore, regarding the relative norms of perceptual and action effector strength, the abstract mathematical concepts of number, addition, and subtraction are similar to the concepts that describe them metaphorically. In other words, relative norms of perceptual and action effector strength of the mathematical concepts of number, addition, and subtraction are similar to the relative norms of the concepts that are used to describe them metaphorically.

**Table 3:** Norms of perceptual strength of numbers, addition, and subtraction (Lynott et al., 2019).

Modality	Auditory	Gustatory	Haptic	Interoceptive	Olfactory	Visual	*M* (SD)
Concept							
Number	1.63	0.11	0.58	0.16	0.05	3.63	1.03 (1.41)
Addition	1.28	0.18	0.50	0.43	0.13	3.33	0.97 (1.22)
Subtraction	2.29	0.06	0.24	0.06	0.00	3.71	1.06 (1.57)
M (SD)	1.73 (0.52)	0.11 (0.06)	0.44 (0.18)	0.21 (0.19)	0.06 (0.06)	3.55 (0.20)	

The last column and row report the means (M) of the means and their standard deviations (SD; in brackets).

**Table 4:** Norms of action effector strength of numbers, addition, and subtraction (Lynott et al., 2019).

Action effector	Foot	Hand	Head	Mouth	Torso	*M* (SD)
Concept						
Number	0.05	0.81	2.57	1.43	0.33	1.04 (1)
Addition	0.37	2.00	3.58	1.37	0.47	1.56 (1.3)
Subtraction	0.00	1.11	3.05	0.42	0.05	0.93 (1.3)
*M* (SD)	0.14 (0.20)	1.31 (0.62)	3.07 (0.50)	1.07 (0.57)	0.29 (0.21)	

The last column and row report the means (M) of the means and their standard deviations (SD; in brackets).

This supports the main assumption of the strong version of embodied cognition, according to which the same sensorimotor networks and resources used to process the base of a metaphor are also used to process the target of that metaphor (Gallese and Lakoff, 2005). In the case of metaphorical description of number, addition, and subtraction in terms of spatial and motion concepts (movement, rightwards, upwards, direction, etc.), the same sensorimotor networks and resources that are employed to process the spatial and motion concepts are also employed to process the concepts of number, addition, and subtraction. In other words, the mathematical concepts of number, addition, and subtraction are grounded in spatial/motion concepts. They are understood through the sensorimotor networks that represent the spatial/motion concepts.

## 5. Perceptual and action effector strength in metaphorical description of the limit of a function

The ideas of embodiment theories and grounded cognition have been used not only in teaching and learning elementary mathematical concepts but also more advanced concepts in mathematics. Some of these higher-level concepts are metaphorically described in mathematical discussions (Glenberg, 2022). In this section, we use Lancaster Sensorimotor Norms to discuss the grounded understanding of limit of function. Limit of a function, which is one of the most fundamental concepts in calculus, is a mathematical concept that is metaphorically described in terms of spatial and motion concepts, although its formal definition is totally based on abstract mathematical symbols. This concept is a base for defining many other concepts in calculus such as continuity, derivative, and integral (e.g., Leithold, 1997). The metaphorical description of this concept as a fictive motion has also been discussed in some works (e.g., Núñez and Lakoff, 1998; Lakoff and Núñez, 2000; Marghetis and Núñez, 2013). According to the formal definition of the limit of a function in mathematics textbooks, the limit of the function *f(x)* at *c* is equal to *L* ( $\underset{x\to c}{\lim}f\left(x\right)=L)$ if:

$$\forall \varepsilon >0,\exists \delta >0,\mathrm{such that if}\;0<\;x\;\;-\;\;\mathrm{c<}\;\;\delta ,\;\;\mathrm{then}\;f\left(x\right)-\mathrm{L<}\;\varepsilon$$

Since acquiring a clear understanding of this definition may be difficult for students, it is metaphorically expressed to make it more understandable. According to the metaphorical definition, when the moving point *x* approaches the fixed point *c*, the moving point *f(x)* approaches the fixed point *L*, and the distance between *f(x)* and *L* becomes smaller than any small distance (Leithold, 1997). This metaphorical definition of limit is totally expressed in terms of spatial and motion concepts (approach, point, distance, etc.). Here, we can see *x* and *f (x)* as two moving objects. As *x* moves toward *c*, *f(x)* moves toward *L* and the distance between *f(x)* and *L* can become smaller than any distance (Marghetis and Núñez, 2013). The derivative of a function at a given point, which is essentially a limit, is also metaphorically described in terms of spatial and motion concepts. In the graphical representation of the concept of derivative of function that includes spatial and motion concepts, the derivative of a function at the point of *c* is understood as the slope of the tangent line at *c*. We scouted Lynott et al.’s (2019) data base and extracted perceptual and action strength ratings of several spatial and motion concepts (APPROACH, NEIGHBORHOOD, MOVING, DISTANCE, LINE, SLOPE) that are used to metaphorically describe the mathematical concepts of limit and derivative of function. Tables 5, 6 show degrees of perceptual and action effector strength of these concepts.

**Table 5:** Norms of perceptual strength of some spatial and motion concepts used to metaphorically describe limit of function and derivative of function (Lynott et al., 2019).

Modality	Auditory	Gustatory	Haptic	Interoceptive	Olfactory	Visual	*M* (SD)
Concept							
Approach	1.94	0.44	1.17	0.89	0.56	2.94	1.32 (0.96)
Neighborhood	2.00	0.00	0.94	0.39	1.11	4.17	1.44 (1.50)
Moving	1.22	0.06	2.06	1.50	0.06	3.89	1.46 (1.43)
Distance	1.76	0.71	1.43	1.29	0.57	3.19	1.49 (0.94)
Line	0.89	0.00	1.22	0.22	0.00	4.44	1.13 (1.70)
Slope	0.18	0.09	1.64	1.14	0.14	3.68	1.14 (1.40)
*M* (SD)	1.33 (0.71)	0.22 (0.29)	1.41 (0.40)	0.90 (0.51)	0.41 (0.43)	3.72 (0.57)	

The last column and row report the means (M) of the means and their standard deviations (SD; in brackets).

**Table 6:** Norms of action effector strength of some spatial and motion concepts used to metaphorically describe limit of function and derivative of function (Lynott et al., 2019).

Action effector	Foot	Hand	Head	Mouth	Torso	*M* (SD)
Concept						
Approach	3.94	1.71	2.18	1.24	1.82	2.18 (1.04)
Neighborhood	1.70	1.40	2.45	1.00	0.70	1.45 (0.68)
Moving	4.36	4.05	3.47	2.82	3.48	3.64 (0.59)
Distance	2.65	1.45	2.40	0.55	0.70	1.55 (0.96)
Line	0.95	0.84	2.00	0.00	0.00	0.76 (0.83)
Slope	2.50	1.27	2.09	0.27	0.82	1.39 (0.91)
*M* (SD)	2.68 (1.30)	1.79 (1.14)	2.43 (0.54)	0.98 (1.01)	1.25 (1.24)	

The last column and row report the means (M) of the means and their standard deviations (SD; in brackets).

The values presented in these tables show that these concepts have high degrees of perceptual and action effector strength. Among ratings of perceptual strength, ratings of visual modality showed the largest effect compared to the norms of other modalities (*RE_visual_* = 0.91, *RE_haptic_* = 0.62, *RE_auditory_* = 0.58, *RE_interoceptive_* = 0.45, *RE_olfactory_* = 0.24, and *RE_gustatory_* = 0.16. All five pairwise comparisons with this modality were *p* < 0.005). Among norms of action effector strength, norms of foot and head showed larger effects than others (*RE_foot_* = 0.70, *RE_head_* = 0.68, *RE_hand_* = 0.49, *RE_torso_* = 0.33, and *RE_mouth_* = 0.28. In this case, though, no pairwise comparison was significant). Therefore, these elements play a more important role in the process of grounding the abstract mathematical concepts of the limit of function and derivative of function in the physical environment through metaphorical descriptions. Based on the assumptions of the strong version of embodiment, when the concepts of limit or derivative of function are metaphorically understood, the sensorimotor networks that are associated with visual modality, foot, hand, and head are actively employed. Although norms of perceptual and action effector strength of these two calculus concepts have not been provided in Lynott et al.’s (2019) data base, we can expect that these concepts have high degrees of visual strength and high degrees of action effector strength in foot, hand, and head.

Limit and derivative are essentially continuous concepts. That is why these concepts can be understood in terms of motion domains. It can be said that there is some kind of isomorphic relationship between these mathematical concepts and spatial/motion domains. In fact, where two domains have some kind of isomorphic relationship with each other, each one can be understood in terms of the other. Two domains are isomorphic when they share some deep similarity. This essential similarity can be the reason behind the similarity of their behaviors in some respects. Since many mathematical concepts are essentially continuous, they can be described in terms of spatial/motion domains because movements are continuous too.

From the perspective of the strong version of embodiment (Gallese and Lakoff, 2005), these mathematical concepts can be understood through the activation of the sensory-motor system. In other words, these concepts can become grounded in sensory-motor experiences when they are understood in terms of motion domains. Using gestures that describe these motion domains can contribute to the grounding of mathematical concepts that are understood in terms of motion domains (Khatin-Zadeh et al., 2021a; Khatin-Zadeh, 2022). In this way, an abstract conceptual entity that does not exist in the physical form is embodied in terms of a physical object and subsequently is grounded in the physical environment. In fact, when abstract mathematical concepts are described in terms of motion domains supported by gestures that describe those motion domains, the abstract concepts can be grounded in the physical environment through the visual and motor systems (Khatin-Zadeh et al., 2022b,c). They can be grounded through the visual system as motion domains have a high degree of imageability. Also, they can be grounded through the motor system as description of motion domains and gesture involves the activation of the motor system. In this way, even highly abstract mathematical concepts can be grounded in the physical environment if they are understood in terms of highly imageable motion domains supported by gestures that involve the activation of the motor system.

Finally, it should be noted that Lancaster Sensorimotor Norms have been obtained on the basis of an on-line judgment task presented to a group of participants. In this task, English speaking participants rated perceptual and action effector strengths of words that were presented to them in lists out of a natural context. As with all subjective rating studies, such tests rely on participants’ overt calculation of their transient mental state rather than directly measuring embodiment of abstract concepts. Since mathematical concepts are often used in the context of mathematical problems, there might be some variations in the perceptual and action effector strengths of these concepts. In other words, perceptual and action effector strength of mathematical concepts may be affected by the context of a mathematical problem. This could limit our interpretation of the data that have been presented in Lancaster Sensorimotor Norms. The effect of context on the perceptual and action effector strength of concepts during metaphorical description of mathematical concepts is a question that can be investigated in future works.

## 6. Conclusion

When an abstract concept is metaphorically described in terms of a concrete concept, perceptual and action effector strength of the base concept of the metaphor plays a key role in the process of grounding the abstract concept in the physical environment. This is particularly the case when this metaphorical description is supported by gestures. Gestures can offer a highly visible description of visual and motoric features of the base domain of a metaphor. In fact, when gestures accompany a metaphorical description, the process of embodiment takes place through two mechanisms: a verbal-based mechanism and a gesture-based mechanism. Through the first mechanism, a concrete concept is mapped into an abstract concept, and perceptual features of the base are attributed to the target. Through the second mechanism, gestures strengthen the involvement of the sensorimotor system in the process of metaphorical description. In fact, the second mechanism strengthen the process of embodiment through active and direct involvement of body parts. The description of abstract mathematical concepts in terms of graphical representations is an interesting scientific case in which an abstract concept is grounded in a concrete concept. The graphical representation may have some degree of perceptual strength. Since the graphical representation can be depicted by gestures, the motor system can be actively employed to ground abstract mathematical concepts in the concrete world when they are described in terms of graphical representations. If understanding an abstract mathematical concept in terms of a graphical representation could involve the employment of the motor system, it can be assumed that factors determining motor strength of a graphical representation play an important role.

## Data availability statement

The original contributions presented in the study are included in the article/Supplementary material, further inquiries can be directed to the corresponding author.

## Ethics statement

Ethical approval was not required for the study involving humans in accordance with the local legislation and institutional requirements. Written informed consent to participate in this study was not required from the participants or the participants’ legal guardians/next of kin in accordance with the national legislation and the institutional requirements.

## Author contributions

OK-Z wrote the first draft of this manuscript. DF, JH, and FM-R commented on it and revised it. All authors contributed to the article and approved the submitted version.

## Conflict of interest

The authors declare that the research was conducted in the absence of any commercial or financial relationships that could be construed as a potential conflict of interest.

## Publisher’s note

All claims expressed in this article are solely those of the authors and do not necessarily represent those of their affiliated organizations, or those of the publisher, the editors and the reviewers. Any product that may be evaluated in this article, or claim that may be made by its manufacturer, is not guaranteed or endorsed by the publisher.
